# Mycobacteriosis in Aquatic Invertebrates: A Review of Its Emergence

**DOI:** 10.3390/microorganisms8081249

**Published:** 2020-08-17

**Authors:** Nadav Davidovich, Danny Morick, Francesca Carella

**Affiliations:** 1 Israeli Veterinary Services, Bet Dagan 5025001, Israel; 2 Department of Marine Biology, Leon H. Charney School of Marine Sciences, University of Haifa, Haifa 3498838, Israel; dmorick@univ.haifa.ac.il; 3 Morris Kahn Marine Research Station, University of Haifa, Haifa 3498838, Israel; 4 Hong Kong Branch of Southern Marine Science and Engineering Guangdong Laboratory (Guangzhou), Hong Kong, China; 5 Department of Biology, University of Naples Federico II, Complesso Universitario di Monte S. Angelo, Via Cinthia, Ed. 7, 80136 Naples, Italy; francesca.carella@unina.it

**Keywords:** aquatic invertebrate, bacteria, mycobacteriosis, *Mycobacterium*, review, zoonotic microorganisms

## Abstract

Mycobacteriosis is a chronic bacterial disease reported in aquatic and terrestrial animals, including humans. The disease affects a wide range of cultured and wild organisms worldwide. Mycobacteriosis is well-known in aquatic vertebrates (e.g., finfish, marine mammals), while in the last few years, reports of its presence in aquatic invertebrates have been on the rise, for both freshwater and marine species. The number of cases is likely to increase as a result of increased awareness, surveillance and availability of diagnostic methods. Domestication of wild aquatic species and the intensification of modern aquaculture are also leading to an increase in the number of reported cases. Moreover, climate changes are affecting fresh and marine aquatic ecosystems. The increasing reports of mycobacteriosis in aquatic invertebrates may also be influenced by global climate warming, which could contribute to the microbes’ development and survival rates, pathogen transmission and host susceptibility. Several species of the genus *Mycobacterium* have been diagnosed in aquatic invertebrates; a few of them are significant due to their wide host spectrum, economic impact in aquaculture, and zoonotic potential. The impact of mycobacteriosis in aquatic invertebrates is probably underestimated, and there is currently no effective treatment other than facility disinfection. In this review, we provide an overview of the diversity of mycobacterial infections reported in molluscs, crustaceans, cnidarians, echinoderms and sponges. We highlight important issues relating to its pathological manifestation, diagnosis and zoonotic considerations.

## 1. Introduction

Mycobacteriosis is a serious disease across many animal species that has been described in the scientific literature since the 1880s. The genus *Mycobacterium* includes more than 190 species, belonging to the family Mycobacteriaceae, class Corynobacteriales, type Actinobacteria, and kingdom Bacteria [1]. It was first proposed in 1896 [2], to include microorganisms considered at that time to be halfway between bacteria and fungi. The non-tuberculous mycobacteria (NTM) include all *Mycobacterium* species that do not cause tuberculosis or leprosy, thus excluding species of the *Mycobacterium tuberculosis* complex as well as *Mycobacterium leprae* [3]. In the last few years, descriptions of diseases in aquatic invertebrates have emerged in both farmed and wild species of economic interest. The World Organization for Animal Health (OIE) lists numerous diseases of crustaceans and molluscs, potentially the most important invertebrates from a commercial standpoint. On the other hand, there are still gaps of knowledge regarding pathogenic-microorganisms and their impacts on other aquatic invertebrates that inhabit the marine benthos and have some commercial importance, such as echinoderms and sponges.

Mycobacteriosis is a chronic progressive disease caused by various acid-fast bacilli (AFB) affecting numerous wild and cultured aquatic invertebrate species worldwide [4,5]. These microorganisms are saprophytes that reside in both soil and fresh and salt water, where they can exist for prolonged periods; they are gradually being recognized as emerging pathogens causing opportunistic infections in humans, farmed species and wildlife [6]. They are transmitted by ingestion, inhalation and inoculation from environmental sources, rather than from person to person [7].

In aquatic environments, reported hosts of mycobacteria are protozoans (including amoebae), molluscs, crustaceans, cnidarians, echinoderms and sponges. The presence of mycobacteria in these species is due to the widespread distribution of mycobacteria in their habitats, in some cases overlapping with human habitats. Marine invertebrates can be infected by mycobacteria through vectors (e.g., protozoans), where they are simply harboured without causing any harm. In other cases, they can lead to mycobacterial infections [8]. These microorganisms can be part of the animal food chain, thus playing a significant role in their transmission. There are reports of filter-feeding species ingesting mycobacteria, which then survive in the digestive tract for long periods, being protected from desiccation, ultraviolet radiation and other external conditions [9].

Contrary to the common manifestation of mycobacteriosis in fish, where gross examination of the infected animal exhibit grayish to white granulomatous nodules in the parenchyma of various internal organs [10], or external signs such as dermal erosions/ulcerations [11], or cutaneous hyperplastic nodules [12], in aquatic invertebrates, in most cases, gross examination of infected animals shows no external signs of the disease. In some cases, external manifestation may be present in the form of multiple, raised, black to brown lesions of the cuticle or internal organs [13,14]; in other cases, for example, in affected Atlantic sea scallop *Placopecten magellanicus* (Gmelin, 1791), visible orange nodules are present in the animals’ adductor muscle [15].

Whether bacteria of the genus *Mycobacterium* cause primary disease in invertebrates is still under debate. In some cases, mycobacterial infections in aquatic invertebrates co-occur with other pathogens. For example, Davidovich et al. [5], described an infection caused by two pathogenic-microorganisms in redclaw crayfish *Cherax quadricarinatus* (von Martens, 1868): *Mycobacterium gordonae* and *Cherax quadricarinatus* bacilliform virus (CqBV). Pen shells *Pinna nobilis* (Linnaeus, 1758) collected in Italy and Spain presented with co-occurrence of *Mycobacterium* sp., the parasite *Haplosporidium pinnae* and other pathogens [16]. Moreover, Prado et al. [17], reported a case of co-infection of *Vibrio mediterranei* and *Mycobacterium* sp. in *P. nobilis*.

## 2. The Immune System of Aquatic Invertebrates and Mycobacteriosis

In both aquatic and terrestrial invertebrates, their tegument (i.e., epidermis, shell, exoskeleton) is the first mechanical barrier to pathogenic microorganisms [18,19]. However, when these mechanical hindrances are damaged, the invading pathogens gain entry into the tissue/body of the organism. The exposure to pathogenic-microorganisms forthwith stimulates the proteolytic pathways, activated by a variety of molecules (e.g., serine protease, 2-mercaptoethanol, trichloroacetic acid, bromelain, and serpin 12) that decrease or eliminate the invading microorganism [20,21]. In the last few decades, the scientific literature has evidenced some remarkable differences in the repertoires of immune mechanisms in invertebrate taxa and the related inflammatory characteristics [22,23,24]. When stimulated, pathogen-associated molecular patterns are activated and the common effector mechanisms of immune responses in all invertebrates and vertebrates, innate receptors which are known as pattern-recognition receptors. This promotes downstream signalling pathways and altered patterns of gene expression that lead to a first defence mechanism which comprises a cell-mediated response against the invading microorganism, as well as the activation of more prolonged defence immune-mechanisms [25]. In vertebrates, as a part of the chronic inflammatory processes, cells of the immune system produce granulomas, consisting of lymphocytes and macrophages, which can potentially differentiate into morpho-functionally different cells (i.e., plasma cells and multinucleated giant cells). Invertebrates do not have this great variety of immune-cells and responses; immunocytes/haemocytes are the only inflammatory effector cells. Moreover, inflammatory responses in invertebrates are classified as infiltrative, nodular, encapsulation-type and phagocytosis, depending on the type and entity of the insult [26,27,28,29]. The prophenoloxidase system plays a key role in the immune defence of cnidarians, such as corals, and arthropods, as it activates the melanin-synthesis pathway, which yields cytotoxic products and melanin deposition [30,31,32]. The prophenoloxidase mechanism is less represented in molluscs, where accumulating transcript and genomic data are revealing gene-encoded elements for pathogen recognition and clearance [33].

In crustaceans, Davidovich et al. [5], reported on a mycobacterial infection in redclaw crayfish *Cherax quadricarinatus* caused by *M. gordonae*. Histopathological examination indicated that the immune system’s reaction to the pathogenic-microorganism consists of melanised haemocytic aggregations (fixed phagocytes) with perivascular cuffing of the haemal spaces in the hepatopancreas and encapsulation reactions. In bivalves, Grimm et al. [15], described a case of mycobacteriosis in Atlantic sea scallop *Placopecten magellanicus* that was characterized by inflammatory nodules; these were most commonly identified macroscopically at the adductor muscle level, but micro- and macroscopic nodules were present in other tissues of at least some infected animals. Moreover, the pen shell *Pinna nobilis* was found to display two haemocyte types, granular and hyaline, along with aggregates of brown cells filled with melanin and lipofuscin [34]. Granular immune cells increased in number and dimension upon mycobacterial infection in an attempt to destroy the microorganism, with each granulocyte in the haemocyte phagosome filled with AFB [4].

## 3. Mycobacteriosis in Molluscs

### 3.1. The Phylum Mollusca

The phylum Mollusca is the second largest (after Arthropoda) group of invertebrates. This phylum is one of the most diversified groups in the animal kingdom, which include >50,000 species, about 30,000 of which are found in the marine environment. Molluscs live mainly in water, however, a significant number of them are also found ashore [35]. Gastropoda is a class of molluscs which includes over 40,000 species: (a) snails with spirally coiled shells, (b) flat-shelled limpets, (c) shell-less sea slugs and (d) terrestrial snails and (e) slugs. Bivalvia is a class of freshwater and marine molluscs that have laterally compressed shape enclosed by a shell consisting of two-valve shells. [36]. Among the bivalves, mussels, oysters, scallops and clams are the most common in aquaculture. Although the class contains a relatively small number of species, about 7500, it elicits substantial interest chiefly because many of its members are eaten by humans in substantial amounts. The size of the mollusc body ranges from several millimetres up to several metres in cephalopods [9]. They feed on vegetables and animals [9]. Molluscs have gained a certain relevance for humans, and are of interest for various reasons: (a) as an economically important food source, including both terrestrial and aquatic species (gastropods, bivalves, cuttlefish, octopus, and squid), (b) as agricultural pests (e.g., slugs) and (c) as intermediate hosts for different types of parasite (e.g., schistosomiasis, which is also known as snail fever or bilharzia).

### 3.2. The Importance of Molluscs for the Global Economy as a Food Source

According to the Food and Agriculture Organization (FAO), global aquaculture production in 2016 included 17.1 million tonnes (USD 29.2 billion) of farmed molluscs [37], made up mainly of marine bivalve molluscs farmed in seas, lagoons and coastal ponds. Although the quantity of marine molluscs produced by the People’s Republic of China dwarfs that of all other producers, several countries in all regions rely rather heavily on mussels, oysters and, to a lesser extent, gastropods such as abalone for their aquaculture production. In 2016, at least 109 species of molluscs were farmed in different countries: cupped oysters (*Crassostrea* spp.) were the main farmed species (28% of the total), Japanese carpet shell (*Ruditapes philippinarum*) was the second-most farmed mollusc (25%), followed by farmed marine molluscs (*Mollusca* spp.) (7%), sea mussels (Mytilidae) (6%), constricted tagelus (*Sinonovacula constricta*) (5%), Pacific cupped oyster (*Crassostrea gigas*) (3%), blood cockle (*Anadara granosa*) (3%), Chilean mussel (*Mytilus chilensis*) (2%) and other mollusc species (amounting to 11%) [37].

### 3.3. Mycobacteriosis in Gastropods

The locomotion of molluscs in their environment may be active or passive. The environment of aquatic invertebrates harbours a rich bacterial flora and bivalves, because of their efficient filter-feeding mechanism, can ingest a variety of microorganisms. A possible route for the transmission of mycobacteria is animal surface and visceral mass [38]. Moreover, molluscs are ectotherms and internal bacteria multiplication depends on the surrounding temperature of the environment. Descriptions of mycobacterial infections in molluscs are still scarce in the literature, with old reports on gastropods from freshwater environments; on the other hand, for bivalves, relatively recent descriptions are emerging, frequently connected to species involved in human disease. The first identification of mycobacteria in molluscs was described by Pan in 1956 [39] (Table 1). Microscopic observation of the freshwater gastropod *Australorbis glabratus* (synonyms: *Biomphalaria glabrata*) (Say, 1818) showed the presence of two different pathogenic-microorganisms responsible for inflammatory lesions and possibly linked to animal mortality. One of these microorganisms was AFB-like and was found in 3 out of 22 (13.6%) of the observed snails. It appeared intracellularly and as haemocyte aggregates, some of which had features resembling the leprosy bacilli (globi). In this case, the rectal ridge, kidney, gut, mantle, foot and ovotestis were affected and no necrosis was observed in the infected specimens.

A few years later, Michelson in 1961 [43] described an experimental infection trial using a *Mycobacterium*-infected colony of two-ridge rams-horn *Helisoma anceps* (Menke, 1830); the source-infected snails had been maintained in his laboratory since 1954 (Table 1). The history of this snail colony was not known, except that the snails had been maintained in a tropical fish aquarium for some years prior to 1954. This pathogenic-microorganism was an AFB, Gram and alcohol-fast positive, measuring 2.5–5.5 µm in length and 0.3–0.4 µm in width. The infection was grossly evident in living snails, particularly in albino strains. The early appearance of infection was linked to prominent clubbing at the tentacle tips, accompanied by localized hyper-pigmentation. Microscopically, the tips of the tentacles showed marked aplasia with large vacuolated areas of increased pigmentation. The outer epithelium, which is normally columnar in this species, appeared squamous and compressed. Loss of cilia frequently occurred. Small, spherical, refractile yellow bodies were detected in the infected tissues. Histopathology revealed that these yellow bodies were always associated with tubercle-like lesions and their presence appeared to be pathognomonic. In longstanding infections, tubercles developed on the surface epithelium with concurrent atrophy of the tentacles. In some cases, infected amoebocytes cells accumulated in small aggregates with other inflammatory characteristic cells and fibroblasts, forming tubercles. In mature tubercles, necrotic areas became evident; these became vacuolated and contained a large conglomerate of mycobacteria.

Beran et al. [44], also reported two atypical mycobacterial agents in the ramshorn snail *Planorbarius corneus* (Linnaeus, 1758). This snail belongs to the family Planorbidae, which is distributed throughout Europe, including Turkey [51,52], and it usually inhabits small transient ponds and streams. Beran et al. [44] studied the distribution of mycobacteria in clinically healthy ornamental fish and their environment, reporting the presence of *Mycobacterium chelonae* and *Mycobacterium fortuitum* (Table 1).

*Mycobacterium ulcerans* is known as the causative agent of Buruli ulcer, one of the most common atypical mycobacterial diseases in humans [53]. This environmental *Mycobacterium* has been found in swamps and wetlands, the usual habitats for aquatic insects implicated in the transmission of this pathogen [45]. The latter authors found that *M. ulcerans* has a strong tendency to form biofilms on plant surfaces and demonstrated that a few aquatic snails may harbour *M. ulcerans* after feeding on aquatic macrophytes on which *M. ulcerans* biofilm had developed. Marsollier et al. [45], collected aquatic snails *Planorbis planorbis* (Linnaeus, 1758) and *Pomacea canaliculata* (Lamarck, 1819) in the Daloa region of Ivory Coast, an area known to be heavily affected by Buruli disease. The authors reported no detection of mycobacterial growth in the aquatic snails, which may be passive hosts. However, after eating experimentally infected snails, the salivary glands of biting naucorid water bugs were found to contain *M. ulcerans*. The authors isolated the bacterium using Löwenstein-Jensen (LJ) selective medium. Polymerase chain reaction (PCR) assay performed on *Mycobacterium* isolates also indicated other mycobacteria: *M. gordonae* and *Mycobacterium szulgai*. In the same year, Kotlowski et al. [46], collected environmental specimens from Buruli ulcer-endemic regions in Benin, Africa. Among the different specimens analysed, *M. ulcerans* DNA was detected in one out of six specimens of the snail *Bulinus senegalensis* (Muller, 1781) by nested-PCR assay (Table 1).

Many aspects of the marsh snail *Biomphalaria glabrata* (Say, 1818) have been studied because of its role as an intermediate host of the trematode parasite *Schistosoma mansoni*. This snail has a wide geographical distribution and low dispersion, and it is easily collected [54]. In 1988, following a routine screening of a laboratory-maintained colony of an albino strain of *B. glabrata*, Bean-Knudsen et al. [47], showed the presence on one animal of yellow pedunculated masses of a few millimetres in size adjacent to the pneumostome and above the base of the tentacle. The animal showed no clinical signs other than mild behavioural weakness. Diseased and healthy specimens from the same tank were sampled for histopathological examination and stained with the routinely used haematoxylin and eosin (H&E) and other stains: Brown–Hopps for Gram staining, acid–Schiff (PAS), and Ziehl–Neelsen (ZN). Microscopy showed that the inflammatory-mass was made up of numerus immune cells forming capsules and fibrous tissue, separated by mesenchymal tissue. Each of these capsules contained large numbers of phagocytic amoebocytes containing granular to rod-shaped eosinophilic material which was stained positively with PAS and ZN staining. The phagocytic cells containing AFB appeared to migrate toward and through the overlying epithelium. Brown–Hopps Gram staining revealed no other microorganisms in the organs of the affected specimens. In addition, tissues form three moribund snails, two clinically normal snails, and an abnormally cloudy egg mass were inoculated onto LJ and Middlebrook 7H10 agar. Mixed cultures containing AFB were then sub-cultured from active Middlebrook 7H10 agar cultures onto LJ medium. Microbiological examination of moribund snail, consistently resulted in isolates of a *Mycobacterium* sp. mixed in culture with several yeast forms (Table 1).

### 3.4. Mycobacteriosis in Bivalves

The eastern oyster *Crassostrea virginica* (Gmelin, 1791) is a species of true oyster native to the eastern seaboard and Gulf of Mexico coast of North America. The earliest description of NTM isolated from bivalves was done in 1975 [48]. The authors isolated and identified numerous species from *C. virginica* collected in Alabama, USA; from nine pooled samples of eastern oysters *M. scrofulaceum*, *M. gordonae*, *M. terrae* complex, *M. parafortuitum* complex were isolated (Table 1).

Later on, Beecham et al. [49], reported a case of mycobacterial infection in a 66-year-old man who had been shucking oysters. He had a swollen left hand with six non-draining nodular lesions along the ulnar palm (Table 1 and Table 4). Biopsy of a palmar lesion revealed AFB, which proved to be *Mycobacterium marinum* on culture. Epidemiological investigation revealed that 6–8 weeks before the onset of symptoms, the patient had harvested and shucked one gallon of oysters *Crassostrea* sp. (Sacco, 1897) from a river inlet while on holiday in Point Comfort, Texas in the USA. A similar case was reported also in France [50]; a 39-year-old female had an un-healing ulcer in her hand, caused by *M. marinum* (Table 1 and Table 4).

The Atlantic sea scallop *P. magellanicus* is an economically important species in offshore fisheries on the east coast of the USA. In 2016, Grimm et al. [15], reported the presence of orange nodular masses of variable size (up to 1 cm in diameter), predominantly in the adductor muscle tissue but also in other organs of animals collected from the coastline of Massachusetts to Maryland (Table 1). Microscopically, these nodular masses, collected from the adductor muscles were the result of a haemocytic response to a central caseous core of necrotic debris combined with some intact haemocytes. This caseous core was surrounded by several layers of round to oval haemocytes, then delineated externally by a layer of squamous appearing haemocytes. Numerous AFB and Gram-positive bacteria were present, both free and within the inner lamina of intact haemocytes surrounding the caseous cores. In most cases, the nodular masses appeared to effectively sequester the pathogenic-microorganism, but in others, they showed inflammatory of haemocytic nature, including myo-necrosis, and mild oedema. Such pathological-foci (50–300 μm) were mostly common in the sinusoids and connective tissues adjacent to the intestinal loops, gastric epithelium and style sac, and could also be detected in the Atlantic sea scallop digestive gland, kidney, gills and mantle. Sections from specimens containing nodular masses in other tissues were examined using ZN staining, showed AFB in the haemocytes. PCR of the 16S gene and the 16S–23S internal transcribed spacer (ITS) region confirmed the presence of *Mycobacterium* sp. (Table 1).

**Figure 1:**
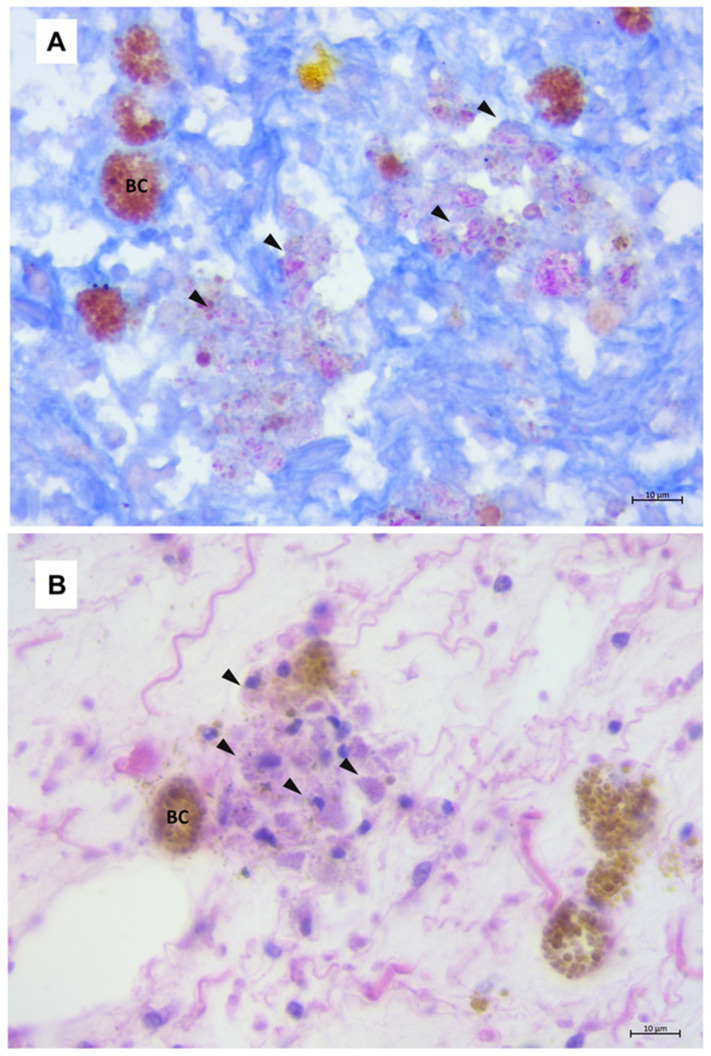
Histopathology of mycobacteriosis in pen shell (*Pinna nobilis*) tissues. (**A**) Infection at the level of the connective tissue circumscribing the digestive gland with immune cell aggregates filled with Ziehl–Neelsen-positive bacteria (arrowheads) and phagocytosed by active brown cells (BC). (**B**) Mycobacteria appear slightly basophilic in haematoxylin and eosin staining.

Recently, Carella et al. [4], described a mycobacterial infection associated with mass mortality events (MME) of the pen shell *P. nobilis* along the coast of Italy, in the Tyrrhenian Sea (part of the Mediterranean Sea) (Table 1). At the time of collection, macroscopically, animals from the Campania region and Sicily showed difficulty closing their valves (gaping) and/or a slow response to touch. The animals also showed diffuse oedema and a retracted mantle. Histopathological examination of all specimens showed varying degrees of inflammatory lesions in the mantle and digestive tissue; depending on the pathology stage and severity, infection was mostly noted in the connective tissue encompassing the gonad and the digestive gland; less frequently, the infection was present in mantle and gills. In two of the examined specimens, necrosis of digestive tubules and gonadal follicles was observed. The inflammation was characterized by large nodular aggregates of immune cells, containing AFB. These cells were observed mainly in the connective tissue encompassing the digestive tissue, gonads, and mantle. Immune cells filled with AFB were also observed in haemolymph vessels; which created aggregate-rich zones coupled with brown cells (Figure 1). Moreover, inflammation of an infiltrative-type was observed around the digestive tubule, where haemocytes containing AFB were noted around the haemolymph vessels. Transmission electron microscopy was also used to assess the features of these microorganisms, which were rod-shaped with a diameter of approximately 0.5 μm and a length of 3.5 μm. PCR of infected animals confirmed that the pathogenic-microorganism was *Mycobacterium*. Blast analysis of amplified sequences from all tested animals revealed that they were grouped with *Mycobacterium sherrisii*, close to the group including *Mycobacterium shigaense*, *Mycobacterium lentiflavum* and *Mycobacterium simiae*. *Mycobacterium* was also reported in other samples from Italy. It was isolated from diseased pen shell specimens from Tuscany, Apulia and Sardinia and in samples from Catalonia in Spain [16]. A molecular study of samples from all of the areas in Italy and Spain using the 65-kDa heat shock protein gene (*hsp65*) and ITS supported the notion that a new species of *Mycobacterium* is infecting *P. nobilis*, close to *Mycobacterium triplex* and belonging to the group of the *M. simiae* complex with *M. sherrisi* [4] (Table 1).

Thereafter, in Greece, two MME of *P. nobilis* were described, in the Gulf of Kalloni and on Lemnos Island [40] (Table 1). Specimens’ histopathology showed the presence rod-shaped, acid-fast, Gram-positive bacilli filling the immune cell constituents, in all collected specimens, resulting in 100% similarity to the report by Carella et al. [4]. *Mycobacterium* was also detected in animals maintained in captivity in the area of Girona and the Ebro Delta (Spain) [17,41]. In both cases, all sequences obtained from sick animals were up to 99% similar to the 16S subunit of *Mycobacterium* sp. available in the NCBI database. The obtained sequences showed an average distance of 0.022. Blast analysis revealed that the sequences from this study grouped together with *Mycobacterium* sp. sequences previously characterized from pen shells [4], green moray (*Gymnothorax funebris*) and spotted moray (*Gymnothorax moringa*) eels [55], and human cases of *Mycobacterium* infections (*M. tuberculosis*, *M. florentinum*, *M. shigaense*, *M. somatepiae*, *M. sherrisii*, *M. genavense*, *M. simiae*, *M. triplex* and *M. lentiflavum*). The phylogenetic analysis showed at least two different groups of sequences in the pen shell phylogenetic tree. Moreover, in Ebro Delta, Prado et al. [17] reported the presence of mycobacterial infection in moribund animals associated with *Vibrio mediterranei* (Table 1).

Recently, MME of *P. nobilis* have been reported, along the Croatian bays of the Adriatic Sea (part of the Mediterranean Sea) [42]. In this survey, the parasite *Haplosporidium pinnae* was identified by histological and molecular methods in all affected specimens, while *Mycobacterium* sp. was detected in some affected and live bivalves. According to the authors, this was the first record of these pathogenic-microorganisms affecting *P. nobilis* in the middle Adriatic Sea (Table 1).

## 4. Mycobacteriosis in Crustaceans

### 4.1. Crustaceans and Their Importance for the Global Economy as a Food Source

Crustaceans belong to the phylum Arthropoda, which is subdivided into four major groups: crustaceans, insects, myriapods, and chelicerates [56]. Crustaceans are considered an important food source worldwide and a few species, such as shrimp, crab and lobster, have become particularly economically important, with some harvested from wild stocks and others being farmed [56]. Crustaceans have a long evolutionary history and remarkable adaptability to different habitats; however, they are not as well studied as their terrestrial arthropod relatives; nevertheless, our knowledge of their behaviour, development physiology and diversity is growing continuously [56].

Global aquaculture production in 2016 included 7.9 million tonnes (USD 57.1 billion) of farmed crustaceans [37]. Marine shrimp, which dominate the production of crustaceans, are typically farmed in coastal aquaculture, and are an important source of foreign exchange earnings for a number of developing countries in Asia and Latin America [37]. In 2016, at least 64 species of crustaceans were farmed in different countries: whiteleg shrimp (*Litopenaeus vannamei*) was the main farmed species (53% of the total), red swamp crayfish (*Procambarus clarkii*) was the second-most farmed crustacean (12%), followed by farmed species of Chinese mitten crab (*Eriocheir sinensis*) (10%), giant tiger prawn (*Penaeus monodon*) (9%), oriental river prawn (*Macrobrachium nipponense*) (3%), giant river prawn (*Macrobrachium rosenbergii*) (3%), and others (10%) [37].

Bacterial infections of crustaceans caused by free-living bacteria may be associated with their exoskeleton [57] or haemolymph [58,59]. In other cases, intracellular bacterial infections occur within specific types of host cells [60,61].

### 4.2. Mycobacteriosis in Crustaceans of the Class Branchiopoda

Crustacean mycobacterial infections are not common and the number of publications describing this pathology are limited. The first report of mycobacteriosis in a crustacean was associated with an atypical mycobacteriosis in humans. Mansson [62], described the case of an aquarium-borne infection caused by *M. marinum* on the lower arm of the aquarium owner (Table 2 and Table 4). The author reported that two black tetra *Gymnocorymbus ternetzi* (Boulenger, 1895) were infected, and that the epidemiological investigations showed that the bacteria had been introduced into the aquarium with common water fleas (probably *Daphnia* sp.); the owner caught the infection while feeding his fish. Soeffing [63] described a mycobacterial infection caused by three different NTM: *M. fortuitum*, *M. chelonae*, and *Mycobacterium flavescens*. The infections were described in *Ceriodaphnia reticulata* (Jurine, 1820), one of the members of the Daphniidae family (Table 2).

The brine shrimp *Artemia* sp. (Leach, 1819) is an important crustacean species in aquaculture because it is highly nutritious. It is also widely used in biological studies because it is easy to culture [64]. Beran et al. [44], investigated the distribution of mycobacteria in clinically healthy ornamental fish and their aquarium environment. They found, among other things, one case of an unidentified *Mycobacterium* in *Artemia salina* (Linnaeus, 1758) (Table 2). In another case, a human cutaneous *M. marinum* infection was reported by LeBlanc et al. [65]. Their investigation of this zoonotic infection from the sea-monkey *Artemia nyos* revealed that the patient later recalled cutting her right third digit while cleaning up a broken glass bowl containing sea-monkeys that had fallen onto her kitchen floor. This event occurred approximately 1 month before the onset of her illness (Table 2 and Table 4).

### 4.3. Mycobacteriosis in Edible Crustaceans

Lightner and Redman [14], also communicated a probable case of *Mycobacterium* sp. infection in whiteleg shrimp *Litopenaeus vannamei* (Boone, 1931). The pathology was detected in a commercial penaeid shrimp hatchery in Florida, USA (Table 2). The population from which the sample came had been collected by a commercial shrimp trawler of the Pacific coast of Panama, and imported to the facility in Florida a few days prior to sampling. One of the three specimens submitted for histopathological screening had multiple grossly visible brown to black pathological-nodules of 1–2 μm in the hepatopancreas and in the tissues that had adhered to that organ during excision from the animal and during fixation and processing. H&E staining revealed that the brown to black nodules were melanised haemocyte nodules. The nodules were present in the hepatopancreas in the intertubular connective tissues and haemocoel, and in the connective tissue capsule of the hepatopancreas in the ovary and the adjacent mandibular organ. The mandibular organ lesion was the most evident. ZN staining confirmed the presence of AFB.

A few decades later, Mohney et al. [66], described mycobacteriosis in the whiteleg shrimp (*L. vannamei*) caused by *Mycobacterium peregrinum*. In this case, multifocal, melanised nodular lesions were observed in the carapace of the cultured marine *L. vannamei*. Macroscopically, infected specimens showed nodular, black, hard-to-friable pathological-lesions, which were located in the subcuticular connective tissue, but did not penetrate into the muscular tissue. Microscopically, infected specimens had multifocal granulomatous lesions within the heart, lymphoid organ, and intertubular connective tissue of the hepatopancreas stained with H&E. Kinyoun acid-fast staining of the tissues revealed clusters of pleomorphic microorganisms that were acid-fast-positive. The bacterial agent was isolated on Middlebrook 7H11 agar and identified by a biochemical test [72], and by high-performance liquid chromatography (HPLC). Another case of mycobacteriosis in juvenile whiteleg shrimp *L. vannamei* (formerly: *Penaeus vannamei*) caused by *M. marinum* was described [67]; the authors reported blackish lesions on the sixth abdominal segment in 20 specimens cultured in Brazil (Table 2).

Brock et al. [13], described a mycobacterial infection in an adult giant freshwater prawn *Macrobrachium rosenbergii* (De Man, 1879) in the USA (Table 2). Macroscopically, the animal showed numerous 1 to 1.5 μm, black to brown, miliary nodules in various tissues: gills, cuticle, abdomen and major organs of the gnathothorax. In this case, pathological-nodules involving the cuticle appeared to have originated from the underlying tissues. Moreover, the specimens’ cuticle was a pale yellowish-green in colour. Areas of opacity were present in the abdominal striated muscle but no other gross pathological signs were noted. The pathogenic-microorganism was isolated and identified as a *Mycobacterium* sp. Microscopically, nodular lesions were mostly found in the heart, antennal gland, hepatopancreas, connective tissue of the gnathothorax, gill stem and lamellae. Some of the nodular-lesions were found in the striated muscle, hindgut submucosa and loose connective tissue of the abdomen. The haemocytic nodules were of different diameter, but typically consisted of a large core of necrotic tissue debris encapsulated by different layers of haemocytes. In all nodules examined, Gram-positive and AFB were present, but were not observed in the cytoplasm of fixed phagocytes. The origin of the mycobacterial infection remained unknown.

In 2010, a mycobacteriosis caused by *M. fortuitum* was described in freshwater red swamp crayfish *Procambarus clarkii* (Girard, 1852) from the Ibrahimiyah Canal in Egypt [68], (Table 2). In this case, 100 animals were examined macroscopically; necrosis of uropods and telsons, congested gills was observed in 11 animals, and 8 individuals showed haemorrhage on the tail-musculature and hepatopancreas tissue. Bacteriological examination produced 14 isolates from the hepatopancreas, which were acid-fast, non-spore-forming and non-motile bacilli to coccobacilli, and suspected of being *Mycobacterium* sp. Molecular examination confirmed that the pathogenic-microorganism was *M. fortuitum*. The authors also conducted an experimental infection and confirmed that it was reproducible. In 2014, Ahmed et al. [69], detected a *Mycobacterium* sp. from 16.6–20.3% of fresh shrimp samples and only 3.7% of frozen shrimp samples in Iraq (Table 2).

**Figure 2:**
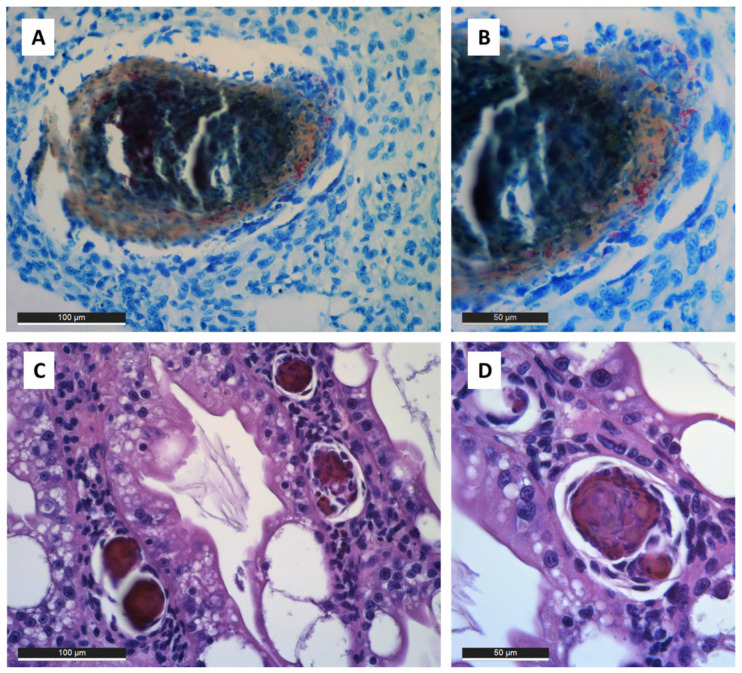
Histopathology of mycobacteriosis in redclaw crayfish (*Cherax quadricarinatus*) tissues. (**A**) Gill tissue with melanised haemocytic aggregation (ZN). (**B**) Detail of haemocytic reaction in the gill with evidence of many magenta-stained acid-fast bacilli attributed to the genus *Mycobacterium* (ZN). (**C**) Melanised haemocytic aggregations in the haemal spaces of the hepatopancreas (H&E). (**D**) Detail of melanised aggregation in the hepatopancreas (H&E). H&E: haematoxylin and eosin; ZN: Ziehl-Neelsen.

Recently, Davidovich et al. [5], described a case of co-infection in redclaw crayfish *C. quadricarinatus* in a small-scale recirculating aquaculture system hatchery in Israel (Table 2). Histopathological examination and bacterial identification confirmed an opportunistic infection caused by *M. gordonae*. Intranuclear inclusion bodies, that were recorded in the tubular epithelium cells of the hepatopancreas by histopathology, indicated co-infection with a viral agent, attributed to CqBV. Histopathology showed melanised haemocytic aggregations with perivascular cuffing of haemal spaces of the hepatopancreas and encapsulation reactions in various tissues (Figure 2). In some melanised aggregates, high numbers of AFB, attributed to the genus *Mycobacterium*, were observed using ZN stain. The bacterial agent was isolated using solid LJ and liquid media (mycobacteria growth indicator tube), and identified with the commercial molecular test GenoType *Mycobacterium* CM.

## 5. Mycobacteriosis in Cnidarians, Echinoderms and Sponges

Cnidaria is a phylum which includes >9000 aquatic invertebrates; their name derived from the presence of cnidocytes (specialized cells) which are connected to supporting cells and neurons. Organisms belonging to this phylum are: (a) corals, (b) hydras, (c) jellyfish, (d) sea anemones, (e) sea pens, (f) Portuguese man-of-war, (g) sea whips, and (h) sea fans. Cnidarians are subdivided into the Anthozoa (Hexacorallia and Octocorallia) with the peculiarity of the lack of a medusa stage, and the Medusozoa, which normally display a medusa stage in their life cycle; this group includes the classes Cubozoa, Staurozoa Hydrozoa, and Scyphozoa, [73].

Only a single publication in the scientific literature has associated a cnidarian species with mycobacteriosis. Smith et al. [74], reported a case of chronic cutaneous *Mycobacterium haemophilum* infection in a human acquired from a coral injury. A 61-year-old previously healthy man developed a subcutaneous nodule in his right arm after scraping a coral while swimming in Thailand, revealing a chronic dermal granuloma linked to the mycobacterial infection (Table 3 and Table 4).

Echinoderms are benthic marine invertebrates living in communities ranging from shallow near-shore waters to the abyssal depths. The nearly 8000 extant echinoderms (from the Greek, meaning “spiny skin”) share several aspects of life cycle and body plan. Five classes are defined: Crinoidea (feather stars and sea lilies), Ophiuroidea (brittle stars), Asteroidea (sea stars and sea daisies), Holothuroidea (sea cucumbers), and Echinoidea (sand dollars and sea urchins) [80]. Existing reports on mycobacterial disease in this group are often related to human disease. A few have reported human cases of atypical mycobacterial infection caused by *M. marinum*, related to injuries from sea urchins in Spain [75,76], and Japan [77], (Table 3 and Table 4). In another case, also resulting from a sea urchin, multiple small, raised nodules over the volar index finger and thumb extending to the palm resulted in tenosynovitis due to a penetrating injury to the hand caused by *M. chelonae* in Hawaii, USA [78], (Table 3 and Table 4).

People coming into contact with sea stars and sea urchins can be injured by their sharp spines or pedicellariae. Acute and chronic reactions to sea urchins have been described. Externally, the latter appear as nodular pathologic-lesions and are designated sea urchin granulomas [75]. The main reports are mostly of the sea urchin *Paracentrotus lividus* (Lamarck, 1816), common in Mediterranean and Atlantic coastal waters. Clinical manifestations after injury by spines of the sea urchin have been reported in France and other parts of Mediterranean [81,82,83,84]. Penetration of the spine into the skin is accompanied by pain, erythema and sometimes edema, which subside after a few hours.

Sponges are members of the phylum Porifera; they are sessile aquatic invertebrates which can be found in freshwater and marine environments. Adult sponges have important ecological role in the aquatic environment (e.g., as filter feeders and bioeroders) and also have economic importance since they have a significant commercial and biopharmaceutical value. Their taxonomy, phylogeny, and evolution, are difficult to reconstruct since many of these them possess only a few systematic and phylogenetic recognizable morphological characters. Moreover, some skeletal characters are prone to homoplasy and are relatively variable with changes of environmental conditions [85].

In the last few decades, marine sponges have been studied intensively, mostly focusing on their microbiomes. However, studies on sponge diseases are still scarce, despite recent attention to associated global population declines of ecologically and commercially important sponge species. The disease epidemics pose serious long-term threats to sponge populations, in particular for the long-lived, slow-growing organisms [86]. Few factors, such as ocean warming, overfishing, acidification, and dominant currents, have been considered relevant to the initial reports of sponge pathologies.

Marine sponges are known to harbour diverse associated bacteria in their tissues, including those of the genus *Mycobacterium*. In 1987, *Mycobacterium poriferae* was described by Padgitt and Moshier [79], who reported its isolation from cell suspensions of the marine sponge *Halichondria bowerbanki* (Burton, 1930) [87,88]. Different species of *Mycobacterium* were isolated from a sponge specimen from the Great Barrier Reef, *Amphimedon queenslandica* (Hooper and Van Soest, 2006), and characterized by sequencing the genes encoding 16S rRNA, the B-subunit of RNA polymerase (*rpoB*), and *hsp65* [88]. These isolates had similarity values of 91.3% to the *rpoB* gene of *Mycobacterium bovis*, *Mycobacterium africanum* and *Mycobacterium parmense*, and 93.1% to the *hsp65* gene of *M. parmense*. Finally, *M. poriferae* was linked to mycobacteriosis in the snakehead fish (*Channa striata*) in Bangkok, Thailand [89].

## 6. Diagnosis of Mycobacteriosis 

Aquatic invertebrate mycobacterioses range from an acute, fulminant disease eventually related to MME [4], to the more frequently observed chronic forms characterized by low bacterial load and chronic inflammation in the host [5]. External clinical signs of disease, if present, are non-specific; they may include multiple raised, black to brown lesions of the cuticle or internal organs [13,14] or visible orange nodules in the adductor muscle as in the case of affected Atlantic sea scallop *P. magellanicus* [15]. Behavioural changes may include lethargy or loss of animals’ equilibrium [5,47]. A number of strategies are used to detect and differentiate mycobacteria in aquatic invertebrate tissues, including histology, culture and molecular methods [47,65]. Histology is the most traditional of these, generally with the detection of AFB using the ZN staining method or modifications thereof. Acid-fast, non-branching bacilli in infected tissue are considered indicative of *Mycobacterium* infections; however, other acid-fast microorganisms (*Nocardia* spp.) may also be present in infected tissue [90]. Mycobacteria are typically cultured on Middlebrook 7H9 broth or 7H10 agar. Tween-20 is used as a carbon source and detergent in the former to prevent clumping. Moreover, LJ selective medium is also frequently employed for mycobacterial culture [65]. Because many NTM infecting aquatic invertebrates are inhibited above 35 °C, and in some cases, at 30 °C, culture at a range of 22–24 °C or at the environmental temperature of the habitat of the invertebrates being studied is recommended. Some isolates are also extremely dysgonic (grow slowly) and relatively poorly on artificial medium, (e.g., *Mycobacterium shottsii*), so extended culture periods (>60 days) should be employed for maximum sensitivity of the test. The culture of NTM from aquatic invertebrates is best performed from internal tissues using aseptic techniques. However, techniques for ‘decontaminating’ samples are available [5]. Molecular methods are increasingly being used to detect *Mycobacterium* in aquatic invertebrates, including varieties of PCR, PCR/RFLP (restriction fragment length polymorphism), qPCR (quantitative PCR) and loop-mediated amplification (LAMP), among others [16,68]. Most of these assays target the genes for 16S or 23S rRNA followed by sequencing techniques. Moreover, the *hsp65* gene encoding a 65 kDa protein is present in all species of mycobacteria and contains epitopes that are unique, as well as some that are common to various species of *Mycobacterium* [91,92]. The *hsp65* gene is known to be more variable than the 16S rRNA gene; hence, it is potentially useful for the identification of genetically related species of bacteria. Furthermore, the ITS gene between the l6S and 23S rRNA genes is roughly from 270 to 360 bp, however, it varies in size among microorganisms. This gene is an appropriate target-gene for probes with which additional phylogenetic data can be accomplished [93]. Moreover, the ITS is suitable gene for differentiating mycobacterial species and can be used to distinguish clinically relevant bacteria subspecies [94].

## 7. Zoonotic Considerations

The essential role of animals in the transmission of infectious diseases has long been recognized. Animals are known to be responsible for the maintenance of infections in nature by harbouring pathogenic-microorganisms, thereby enabling them to survive. In addition, they are actively responsible for pathogen propagation in their environment and the consequent infection of other organisms, including humans [9]. Aquatic invertebrates’ pathogenic mycobacteria all belong to the NTM, also known as atypical mycobacteria. A few species can infect humans, usually causing localized, non-healing wounds in the body extremities [70,75] that may be difficult to treat because of the resistance of some isolates to antimicrobial drugs [65] (Table 4). Our current knowledge of mycobacteriosis due to NTM infection is based on fragmented information from different countries, suggesting that NTM infections are becoming more prevalent.

The ubiquity of aquatic invertebrate mycobacterioses coupled with the apparently low numbers of human cases suggest that, fortunately, the risk to healthy humans is moderate compared to infections caused by the *M. tuberculosis* complex as well as *M. leprae*. However, a small but significant number of persistent infections by atypical mycobacteria have been reported in humans due to trauma followed by exposure to infected surfaces, or in immunosuppressed individuals (Table 4). These infections often require lengthy systemic antibiotic treatment and surgical debridement [78] (Table 4). In a clinical-pathological study, De La Torre et al. [75], reported on ca. 50 biopsy specimens from 35 patients diagnosed as having sea urchin granuloma. Half of these patients were involved in fishing activities, and 50% of these were divers involved in the commercial harvesting of sea urchins. Later, López Zabala et al. [76], reported a case of osteomyelitis of the first metatarsal bone after accidental puncture injury by a sea urchin in a 44-year-old female non-immunosuppressed patient presenting with a 1-month history of pain and swelling of the hallux of the left foot, requiring surgical treatment (Table 4).

In the case of human mycobacterial infections related to crustaceans, there are few reports: (a) Mansson [62], describing an aquarium-borne infection on the owner’s lower arm; (b) LeBlanc et al. [65], described another case of mycobacteriosis related to crustaceans in Pennsylvania, USA (Table 4). In this case, a 43-year-old female had five nodular lesions on the right hand and forearm: one at the base of the nail of the right third digit, one in the region of the third metacarpophalangeal joint and three along the right forearm. The infection was caused by *M. marinum* while cleaning up a broken glass bowl containing sea-monkeys *Artemia nyos* that had fallen onto her kitchen floor (Table 4); (c) another case, described by Jernigan and Farr [70], presented lesions that revealed granulomatous inflammation caused by *M. marinum*. The patient had been pinched on the right fourth finger by a crab while fishing on the South Carolina coast, USA (Table 4). The pathogenic-microorganism deeply penetrated the skin and the proximal fingernail bed on the dorsal aspect of the finger; (d) a few years ago, Lee et al. [71], described a case of tenosynovitis in a 56-year-old woman with a history of puncture injury to the finger caused by a crab. In this case, the patient presented symptoms of pain, redness, and swelling in the second and fifth fingers of the right hand. One year previously, the lady had suffered from a puncture injury her hand caused by a crab; the injured finger showed swelling and redness 2 months later and she waited 10 months before seeking treatment (Table 2 and Table 4).

## 8. Conclusions

Mycobacteriosis occurs in aquatic invertebrates living mostly at tropical and temperate latitudes, and has been reported in wild, cultured and ornamental species. The emergence of mycobacteriosis in different species of aquatic invertebrates in the last few years may be linked to environmental stressors. Indeed, climate changes are affecting the marine ecosystem; in particular, climate warming can increase microorganisms’ development and survival rates, pathogen transmission and host susceptibility [95]. It has been recently reported that even deep-ocean biodiversity is becoming exposed to climate-warming effects [96].

Unlike homeotherms (i.e., mammals and birds) that regulate their internal environments, aquatic invertebrates are poikilotherms which depend on water temperature, with no ability to regulate their core body temperature. Aquatic invertebrates have to survive and adapt to environmental stresses. Temperature change may lead to changes in membrane lipid ordering and, in the case of elevated temperatures, may lead to protein unfolding and denaturation. If these alterations are not corrected rapidly, the functions, and possibly survival of the stressed organism will be at high risk [97]. In this situation, both the pathogen and the host are physiologically tied to the environment in which they live and have an optimal temperature range for survival. In cultured aquatic invertebrates, disease outbreaks are often related to husbandry factors such as poor water quality, inadequate water temperature, high stocking density or handling [5,63].

Numerous species of the genus *Mycobacterium* have been named as etiological agents of aquatic invertebrate mycobacterioses. The first description in molluscs was in 1956 by Pan [39], describing an infection caused by an unidentified species of *Mycobacterium* in the snail *Australorbis glabratus*. Since that first description, publications describing mycobacteriosis in molluscs have accumulated (Table 1); in the last few years, separate cases of mycobacteriosis have been described in the edible pen shell (*Pinna nobilis*) from the Mediterranean coast of Italy, Spain, Greece and Croatia [4,16,17,40,41,42].

The first case of a mycobacterial infection in crustaceans was reported in a small planktonic crustacean, the common water flea (*Daphnia* sp.) from a domestic aquarium; the infectious agent in this case was *M. marinum* [62]. Over the years, mycobacteriosis in crustaceans has been reported in both freshwater species, for example *M. gordonae* in redclaw crayfish *C. quadricarinatus* [5], and saltwater species, for example *M. marinum*, *M. peregrinum*, and a *Mycobacterium* sp. in the edible whiteleg shrimp *L. vannamei* [14,63,65] (Table 2).

The mycobacterial species that is most commonly reported as a pathogen of aquatic invertebrates is *M. marinum*, which is also the most common etiological agent of fish mycobacteriosis [12]. Despite its name, *M. marinum* infects both freshwater and marine aquatic invertebrates in tropical and temperate waters, and is the most widely reported mycobacterial pathogen of aquatic invertebrates (Table 1, Table 2 and Table 3).

Diagnosis of mycobacteriosis in aquatic invertebrates (as in other animal species including humans), has become more accurate due to the development of new detection methods based on new technologies, such as HPLC, as well as amplification of specific gene fragments by PCR assay followed by restriction analysis, as described for the analysis of gene regions such as *hsp65* and rRNA [98]. Among the most important and specific approaches that is mainly applicable, is amplification of 16S rRNA followed by sequencing [1].

Human infections caused by NTM are typically confined to cutaneous lesions in limbs, although deep tissue infections of the musculature, tendons and bones have been also reported, and systemic infections may occur on rare occasions in immunocompromised individuals. Handling infected marine species is the main source of infection for man, especially aquarists, aquafarmers, fishermen and people who work in the processing of aquatic invertebrates, and they should be cautioned about contacting potentially infected invertebrates or fomites.

## Figures and Tables

**Table 1:** Summary of molluscs’ mycobacterioses, including animal species, *Mycobacterium* species, infected tissue, geographical location, and references.

Animal Species	*Mycobacterium* Species	Infected Tissue	Geographical Location	Reference
Pen shell (*Pinna nobilis*)	*M. sherrisi* (*M. triplex*), *Mycobacterium* sp.	Connective tissue surrounding the digestive gland, mantle interstitium, and kidney	Italy, Spain, Greece and Croatia	[4,16,17,40,41,42]
Atlantic sea scallop (*Placopecten magellanicus*)	*Mycobacterium* sp.	Adductor muscle, digestive gland, kidney, gills and mantle	Massachusetts to Maryland, USA	[15]
*Australorbis glabratus*	*Mycobacterium* sp.	Rectal ridge, kidney, gut, mantle, foot and gonad	nr	[39]
Two-ridge rams-horn (*Helisoma anceps*)	*Mycobacterium* sp.	Surface epithelium, connective tissue surrounding the digestive gland, kidney, rectal ridge and foot	USA	[43]
Great ramshorn (*Planorbarius corneus*)	*M. chelonae*, *M. fortuitum*	nr	Czech Republic	[44]
*Planorbis planorbis*, *Pomacea canaliculata*	*M. ulcerans*, *M. gordonae*, *M. szulgai*	Digestive tract	Daloa region of Ivory Coast	[45]
*Bulinus senegalensis*	*M. ulcerans*	nr	Benin	[46]
Marsh snail (*Biomphalaria glabrata*)	*Mycobacterium* sp.	Integument, pneumostome, base of the tentacle, digestive and genital epithelia	USA	[47]
Eastern oyster (*Crassostrea virginica*)	*M. scrofulaceum*, *M. gordonae*, *M. terrae* complex, *M. parafortuitum* complex	nr	Alabama, USA	[48]
Oysters (*Crassostrea* sp.)	*M. marinum*	nr	USA, France	[49,50]

nr, not reported.

**Table 2:** Summary of crustaceans’ mycobacterioses, including animal species, *Mycobacterium* species, infected tissue, geographical location, and references.

Animal Species	*Mycobacterium* Species	Infected Tissue	Geographical Location	Reference
Redclaw crayfish (*Cherax quadricarinatus*)	*M. gordonae*	Hepatopancreas, gills, testis	Israel	[5]
Giant freshwater prawn (*Macrobrachium rosenbergii*)	*Mycobacterium* sp.	Hepatopancreas, heart, antennal gland, loose connective tissue of the gnathothorax, gills	USA	[13]
Whiteleg shrimp (*Litopenaeus vannamei*)	*Mycobacterium* sp., *M. peregrinum*, *M. marinum*	Mandibular organ and hepatopancreas; carapace and heart; carapace and muscle	USA, Brazil	[14,66,67]
Brine shrimp (*Artemia salina*)	*Mycobacterium* sp.	nr	Czech Republic	[44]
Common water flea (*Daphnia* sp.)	*M. marinum*	nr	Sweden	[62]
*Ceriodaphnia reticulata*	*M. fortuitum*, *M. chelonae*, *M. flavescens*	nr	Germany	[63]
Sea-monkey (*Artemia nyos*)	*M. marinum*	nr	Pennsylvania, USA	[65]
Red swamp crayfish (*Procambarus clarkii*)	*M. fortuitum*	Hepatopancreas	Ibrahimiyah Canal, Egypt	[68]
Shrimp	*Mycobacterium* sp.	nr	Iraq	[69]
Crab	*M. marinum*	nr	South Carolina coast, USA	[70]
Crab	*M. arupense*	nr	Republic of Korea	[71]

nr, not reported.

**Table 3:** Summary of echinoderm, cnidarian and sponge mycobacterioses, including animal species, *Mycobacterium* species, infected tissue, geographical location, and references.

Animal Species	*Mycobacterium* Species	Infected Tissue	Geographical Location	Reference
Coral	*M. haemophilum*	nr	Thailand	[74]
Sea urchin (*Paracentrotus* *lividus*)	*M. marinum*	nr	Spain	[75]
Sea urchin	*M. marinum*	nr	Spain	[76]
Sea urchin	*M. marinum*	nr	USA	[77]
Sea urchin	*M. chelonae*	nr	Hawaii, USA	[78]
Sponge	*M. poriferae*	nr	USA	[79]

nr, not reported.

**Table 4:** Summary of human mycobacterioses related to aquatic invertebrates.

Animal Species	*Mycobacterium* Species	Human Injury	Country	Reference
Oysters (*Crassostrea* sp.)	*M. marinum*	A swollen left hand with six non-draining nodular lesions along the ulnar palm.	USA	[49]
Oysters (*Crassostrea* sp.)	*M. marinum*	Ulcer on the hand.	France	[50]
Common water fleas (*Daphnia* sp.)	*M. marinum*	Aquarium-borne infection on the lower arm of the owner.	Sweden	[62]
Sea-monkey (*Artemia nyos*)	*M. marinum*	Five nodular lesions on the right hand and forearm. One at the base of the nail of the right third digit, one in the region of the third metacarpophalangeal joint and three along the right forearm.	Pennsylvania, USA	[65]
Coral	*M. haemophilum*	A chronic cutaneous lesion in an immunologically normal patient after a coral injury, which implicated coral.	Thailand	[74]
Sea urchin (*Paracentrotus* *lividus*)	*M. marinum*	In a study of 50 biopsy specimens from 35 patients diagnosed as having sea urchin granuloma. Half of these patients were involved in fishing activities, and 50% of these were divers involved in commercial harvesting of sea urchins.	Spain	[75]
Sea urchin	*M. marinum*	Osteomyelitis of the first metatarsal bone, after accidental puncture injury by a sea urchin requiring surgical treatment in a non-immunosuppressed patient.	Spain	[76]
Sea urchin	*M. marinum*	Arthritis in the interphalangeal joint of the hallux while snorkelling in Japan.	Fukuoka, Japan	[77]
Sea urchin	*M. chelonae*	Multiple small, raised nodules over the volar index finger and thumb extending to the palm and resulting in tenosynovitis due to penetrating injury by a sea urchin to the hand.	Hawaii, USA	[78]
Crab	*M. marinum*	Granulomatous inflammation on the right fourth finger while fishing.	South Carolina coast, USA	[70]
Crab	*M. arupense*	Tenosynovitis in a patient with a history of puncture injury to the finger.	Republic of Korea	[71]

nr, not reported.

## References

[1] Tortoli E., Velayati A.A., Farnia P. (2019). The taxonomy of the genus *Mycobacterium*. Nontuberculous Mycobacteria (NTM).

[2] Lehmann K.B., Neuman R. (1896). Atlas und Grundriss der Bakteriologie und Lehrbuch der Speziellen Bakteriologischen Disagnostik.

[3] Fedrizzi T., Meehan C.J., Grottola A., Giacobazzi E., Fregni Serpini G., Tagliazucchi S., Fabio A., Bettua C., Bertorelli R., De Sanctis V. (2017). Genomic characterization of nontuberculous mycobacteria. Sci. Rep..

[4] Carella F., Aceto S., Pollaro F., Miccio A., Iaria C., Carrasco P.P., Prado P., De Vico G. (2019). A mycobacterial disease is associated with the silent mass mortality of the pen shell *Pinna nobilis* along the Tyrrhenian coastline of Italy. Sci. Rep..

[5] Davidovich N., Pretto T., Blum S.E., Baider Z., Grossman R., Kaidar-Shwartz H., Dveyrin Z., Rorman E. (2019). *Mycobacterium gordonae* infecting redclaw crayfish *Cherax quadricarinatus*. Dis. Aquat. Organ..

[6] Kazda J., Falkinham J.O., Pavlik I., Hruska K. (2009). The Ecology of Mycobacteria: Impact on Animal’s and Human’s Health.

[7] Falkinham J.O. (1996). Epidemiology of infection by nontuberculous mycobacteria. Clin. Microbiol. Rev..

[8] Vaerewijck M.J.M., Huys G., Palomino J.C., Swings J., Portaels F. (2005). Mycobacteria in drinking water distribution systems: Ecology and significance for human health. FEMS Microbiol. Rev..

[9] Pavlik I., Falkinham J.O., Kazda J., Pavlik I., Falkinham J.O., Hruska K. (2009). The occurrence of pathogenic and potentially pathogenic mycobacteria in animals and the role of the environment in the spread of infection. The Ecology of Mycobacteria: Impact on Animal’s and Human’s Health.

[10] Zanoni R.G., Florio D., Fioravanti M.L., Rossi M., Prearo M. (2008). Occurrence of *Mycobacterium* spp. in ornamental fish in Italy. J. Fish. Dis..

[11] Astrofsky K.M., Schrenzel M.D., Bullis R.A., Smolowitz R.M., Fox J.G. (2000). Diagnosis and management of atypical *Mycobacterium* spp. infections in established laboratory zebrafish (*Brachydanio rerio*) facilities. Comp. Med..

[12] Davidovich N., Pretto T., Sharon G., Zilberg D., Blum S.E., Baider Z., Edery N., Morick D., Grossman R., Shwartz H.K. (2020). Cutaneous appearance of mycobacteriosis caused by *Mycobacterium marinum*, affecting gilthead seabream (*Sparus aurata*) cultured in recirculating aquaculture systems. Aquaculture.

[13] Brock J.A., Nakagawa L.K., Shimojo R.J. (1986). Infection of a cultured freshwater prawn, *Macrobrachium rosenbergii* de Man (Crustacea: Decapoda), by *Mycobacterium* spp., Runyon Group II. J. Fish. Dis..

[14] Lightner D.V., Redman R.M. (1986). A probable *Mycobacterium* sp. infection of the marine shrimp *Penaeus vannamei* (Crustacea: Decapoda). J. Fish. Dis..

[15] Grimm C., Huntsberger C., Markey K., Inglis S., Smolowitz R. (2016). Identification of a *Mycobacterium* sp. as the causative agent of orange nodular lesions in the Atlantic sea scallop *Placopecten magellanicus*. Dis. Aquat. Organ..

[16] Carella F., Antuofermo E., Farina S., Salati F., Mandas D., Prado P., Panarese R., Marino F., Fiocchi E., Pretto T. (2020). In the wake of the ongoing mass mortality events: Co-occurrence of *Mycobacterium*, *Haplosporidium* and other pathogens in *Pinna nobilis* collected in Italy and Spain (Mediterranean Sea). Front. Mar. Sci..

[17] Prado P., Catanese G., Jofre A.G., Andree K.B., Garcia March J.R., Cabanes P., Carella F., Garcia-March J.R., Tena J., Roque A. (2020). Presence of *Vibrio mediterranei* associated to major mortality in stabled individuals of *Pinna nobilis* L.. Aquaculture.

[18] Söderhäll K., Smith V.J., Brehélin M. (1986). The prophenoloxidase activating system: The biochemistry of its activation and role in arthropod cellular immunity, with special reference to crustaceans. Immunity in Invertebrates.

[19] Abbas M.N., Kausar S., Sun Y.-X., Sun Y., Wang L., Qian C., Wei G.-Q., Zhu B.-J., Liu C.-L. (2017). Molecular cloning, expression, and characterization of E2F Transcription Factor 4 from *Antheraea pernyi*. Bull. Entomol. Res..

[20] Ratcliffe N.A., Rowley A.F., Fitzgerald S.W., Rhodes C.P. (1985). Invertebrate immunity: Basic concepts and recent advances. Int. Rev. Cytol..

[21] Kausar S., Abbas M.N., Qian C., Zhu B., Gao J., Sun Y., Wang L., Wei G., Liu C. (2018). Role of *Antheraea pernyi* serpin 12 in prophenoloxidase activation and immune responses. Arch. Insect Biochem. Phys..

[22] Ghosh J., Man Lun C., Majeske A.J., Sacchi S., Schrankel C.S., Courtney Smith L. (2011). Invertebrate immune diversity. Dev. Comp. Immun..

[23] Cerenius L., Söderhäll K. (2013). Variable immune molecules in invertebrates. J. Exp. Biol..

[24] Abbas M.N., Kausar S., Cui H. (2019). The biological role of peroxiredoxins in innate immune responses of aquatic invertebrates. Fish. Shellfish Immunol..

[25] Herrin B.R., Coope M.D. (2010). Alternative adaptive immunity in jawless vertebrates. J. Immunol..

[26] Tripp M.R. (1960). Biological Bulletin.

[27] Smith V.J., Chisholm J.R.S., Beck G., Sugumaran M., Cooper E.L. (2001). Antimicrobial proteins in crustaceans. Phylogenetic Perspectives on the Vertebrate Immune System.

[28] De Vico G., Carella F. (2012). Morphological features of the inflammatory response in molluscs. Res. Vet. Sci..

[29] Kausar S., Abbas M.N., Qian C., Zhu B., Sun Y., Sun Y., Wang L., Wei G., Maqsood I., Liu C.-L. (2017). Serpin-14 negatively regulates prophenoloxidase activation and expression of antimicrobial peptides in Chinese oak silkworm *Antheraea pernyi*. Dev. Comp. Immunol..

[30] Kawabata S., Muta T., Iwanaga S., Söderhäll K., Iwanaga S., Vasta G.R. (1996). Clotting cascade and defense molecules found in the hemolymph of the horseshoe crab. New Directions in Invertebrate Immunology.

[31] Vargas-Albores F., Yepiz-Plascencia G. (2000). Beta glucan binding protein and its role in shrimp immune response. Aquaculture.

[32] Abbas M.N., Kausar S., Sun Y.-X., Tian J.W., Zhu B.-J., Liu C.-L. (2018). Suppressor of Cytokine Signaling 6 can enhance epidermal growth factor receptor signaling pathway in *Bombyx mori* (Dazao). Dev. Comp. Immnol..

[33] Gerdol M. (2017). Immune-related genes in gastropods and bivalves: A comparative overview. Invertebr. Surv. J..

[34] Matozzo V., Pagano M., Spinelli A., Caicci F., Faggio C. (2016). *Pinna nobilis*: A big bivalve with big haemocytes?. Fish. Shellfish Immunol..

[35] Parkhaev P.Y. (2017). Origin and the early evolution of the phylum Mollusca. Paleontol. J..

[36] Pyron M., Brown K.M., Thorp J.H., Rogers D.C. (2015). Introduction to Mollusca and the class Gastropoda. Thorp and Covich’s Freshwater Invertebrates.

[37] FAO (2018). The State of Fisheries and Aquaculture in the World 2018.

[38] Kazda J., Pavlik I., Falkinham J.O., Hruska K. (2009). The chronology of *Mycobacteria* and the development of mycobacterial ecology. The Ecology of Mycobacteria: Impact on Animal’s and Human’s Health.

[39] Pan C. (1956). Studies on the biological control of schistosome-bearing snails: A preliminary report on pathogenic microorganisms found in *Australorbis glabratus*. J. Parasit..

[40] Lattos A., Giantsis I.A., Karagiannis D., Michaelidis B. (2020). First detection of the invasive haplosporidian and mycobacteria parasites hosting the endangered bivalve *Pinna nobilis* in Thermaikos Gulf, North Greece. Mar. Environ. Res..

[41] Lopez-Sanmartin M., Lopez Fernandez J.R., de la Herran R., Garcia March J.R., Navas J.I. Evidence of mycobacterial presence in *Pinna nobilis* infected by *Haplosporidium pinnae* maintained under quarantine conditions. Proceedings of the II Congreso de Juvenes Investigadores del Mar, 2018–2020.

[42] Čižmek H., Čolić B., Gračan R., Grau A., Catanese G. (2020). An emergency situation for pen shells in the Mediterranean: The Adriatic Sea, one of the last *Pinna nobilis* shelters, is now affected by a mass mortality event. J. Invertebr. Pathol..

[43] Michelson E.H. (1961). An acid-fast pathogen of fresh-water snails. Am. J. Trop. Med. Hyg..

[44] Beran V., Matlova L., Dvorska L., Svastova P., Pavlik I. (2006). Distribution of mycobacteria in clinically healthy ornamental fish and their aquarium environment. J. Fish. Dis..

[45] Marsollier L., Sévérin T., Aubry J., Merritt R.W., Saint André J.-P., Legras P., Manceau A.-L., Chauty A., Carbonnelle B., Cole S.T. (2004). Aquatic snails, passive hosts of *Mycobacterium ulcerans*. Appl. Environ. Microbiol..

[46] Kotlowski R., Martin A., Ablordey A., Chemlal K., Fonteyne P.-A., Portaels F. (2004). One-tube cell lysis and DNA extraction procedure for PCR-based detection of *Mycobacterium ulcerans* in aquatic insects, molluscs and fish. J. Med. Microbiol..

[47] Bean-Knudsen D.E., Uhazy L.S., Wagner J.E., Young B.M. (1988). Systemic infection of laboratory-reared *Biomphalaria glabrata* (Mollusca: Gastropoda) with an acid-fast bacillus. J. Invertebr. Pathol..

[48] Hosty T.S., McDurmont C.I. (1975). Isolation of acid-fast organisms from milk and oysters. Health Lab. Sci..

[49] Beecham H.J., Oldfield E.C., Lewis D.E., Buker J.L. (1991). *Mycobacterium marinum* infection from shucking oysters. Lancet.

[50] Aubry A., Chosidow O., Caumes E., Robert J., Cambau E. (2002). Sixty-three cases of *Mycobacterium marinum* infection. Arch. Int. Med..

[51] Hyman L.H. (1967). The Invertebrates. Mollusca I..

[52] Sesen R., Yildirim M.Z. (1993). A study on Turkish freshwater snails that have parasitological importance. Türk. Parazitol. Derg..

[53] Coudereau C., Besnard A., Robbe-Saule M., Bris C., Kempf M., Johnson R.C., Brou T.Y., Gnimavo R., Eyangoh S., Khater F. (2020). Stable and local reservoirs of *Mycobacterium ulcerans* inferred from the nonrandom distribution of bacterial genotypes, Benin. Emerg. Inf. Dis..

[54] De Freitas Tallarico L., Borrely S.I., Hamada N., Siqueira Grazeffe V., Pires Ohlweiler F., Okazaki K., Granatelli A.T., Pereira I.W., de Braganca Pereira C.A., Nakano E. (2014). Developmental toxicity, acute toxicity and mutagenicity testing in freshwater snails *Biomphalaria glabrata* (Mollusca: Gastropoda) exposed to chromium and water samples. Ecotoxicol. Environ. Saf..

[55] Levi M.H., Bartell J., Gandolfo L., Smole S.C., Costa S.F., Weiss L.M., Johnson L.K., Osterhout G., Herbst L.H. (2003). Characterization of *Mycobacterium montefiorense* sp. nov., a novel pathogenic mycobacterium from moray eels that is related to *Mycobacterium triplex*. J. Clin. Microbiol..

[56] Vanhook A.M., Patel N.H. (2008). Crustaceans. Curr. Biol..

[57] Getchell R.G. (1989). Bacterial shell disease in crustaceans: A review. J. Shellfish Res..

[58] Tubiash H.S., Sizemore R.K., Colwell R.R. (1975). Bacterial flora of the hemolymph of the blue crab, *Callinectes sapidus*: Most probable numbers. App. Environ. Microbiol..

[59] Wang W. (2011). Bacterial diseases of crabs: A review. J. Invertebr. Pathol..

[60] Vincent A.G., Breland V.M., Lotz J.M. (2004). Experimental infection of Pacific white shrimp *Litopenaeus vannamei* with necrotizing hepto-pancreatitis (NHP) bacterium by per os exposure. Dis. Aquat. Org..

[61] Eddy F., Powell A., Gregory S., Nunan L.M., Lightner D.V., Dyson P.J., Rowley A.F., Shields R.J. (2007). A novel bacterial disease of the European shore crab, *Carcinus maenas*—Molecular pathology and epidemiology. Microbiology.

[62] Mansson T. (1970). Mycobacteria from aquaria. Br. Med. J..

[63] Soeffing K. (1990). Verhaltensökologie der Libelle *Leucorrhinia rubicunda* L. unter Besonderer Berücksichtingung NahrungsöKologischer Aspekte. Dissertation.

[64] Asem A., Rastegar-Pouyani N., De Los Ríos-Escalante P. (2010). The genus Artemia leach, 1819 (Crustacea: Branchiopoda). I. True and false taxonomical descriptions. Lat. Am. J. Aquat. Res..

[65] LeBlanc J., Webster D., Tyrrell G.J., Chiu I. (2012). *Mycobacterium marinum* infection from sea monkeys. Can. J. Inf. Dis. Med. Microbiol..

[66] Mohney L.L., Poulos B.T., Brooker J.H., Cage G.D., Lightner D.V. (1998). Isolation and identification of *Mycobacterium peregrinum* from the Pacific white shrimp *Penaeus vannamei*. J. Aquat. Anim. Health.

[67] Pedrosa V.F., Wasielesky Júnior W., Klosterhoff M.C., Romano L.A., de Lara G.R. (2017). Micobacteriose Em Camarão Branco Do Pacífico, *Litopenaeus vannamei*. Bol. Instit. Pesca.

[68] Ahmed S.M., Saad El-deen A.G., Elkamel A.A., Mohamed A.M. (2010). Mycobacteriosis in fresh water crayfish (*Procambarus clarkii*). Proceedings of the 14th Science Congress, Faculty of Veterinary Medicine.

[69] Ahmed W.A., Al-gburi N.M., Abbas M. (2014). Incidence of *Mycobacteria* spp. in shrimp in Iraq. MRVSA.

[70] Jernigan J.A., Farr B.M. (2000). Incubation period and sources of exposure for cutaneous *Mycobacterium marinum* infection: Case report and review of the literature. Clin. Infect. Dis..

[71] Lee S.J., Hong S.K., Park S.S., Kim E.-C. (2014). First Korean case of *Mycobacterium arupense* tenosynovitis. Ann. Lab. Med..

[72] Kent P.T., Kubica G.P. (1985). Public Health Mycobacteriology. A Guide for the Level III Laboratory.

[73] Boero F., Bouillon J., Piraino S. (2005). The role of cnidaria in evolution and ecology. Ital. J. Zool..

[74] Smith S., Taylor G.D., Fanning E.A. (2003). Chronic cutaneous *Mycobacterium haemophilum* infection acquired from coral injury. Clin. Infect. Dis..

[75] De La Torre C., Vega A., Carracedo A., Toribio J. (2001). Identification of *Mycobacterium marinum* in sea-urchin granulomas. Br. J. Dermatol..

[76] López Zabala I., Poggio Cano D., García-Elvira R., Asunción Márquez J. (2012). *Mycobacterium marinum* osteomyelitis of the first metatarsal. Eur. J. Orthop. Surg. Trauma.

[77] Schefflein J., Umans H., Ellenbogen D., Abadi M. (2012). Sea urchin spine arthritis in the foot. Skelet. Radiol..

[78] Vargas C.R., Kanwar A., Dousa K.M., Skalweit M.J., Rowe D., Gatherwright J. (2018). Mycobacterial tenosynovitis after sea urchin spine injury in an immunocompromised patient. Open Infect. Dis..

[79] Padgitt P.J., Moshier S.E. (1987). *Mycobacterium poriferae* sp. nov., a scotochromogenic, rapidly growing species isolated from a marine sponge. Int. J. Syst. Bacteriol..

[80] Smith L.C., Arizza V., Barela Hudgell M.A., Barone G., Bodnar A.G., Buckley K.M., Cunsolo V., Dheilly N.M., Franchi N., Fugmann S.D., Cooper E.L. (2018). Echinodermata: The complex immune system in echinoderms. Advances in Comparative Immunology.

[81] Gaté J., Cuilleret P., Chanial G., Bouquin H. (1936). Lésions papulo-nécrotiques à réaction histologique tuberculoïde dues à l’inclusion d’épines d’oursins. Bull. Soc. Fr. Dermatol. Syphiligr..

[82] Rocha G., Fraga S. (1962). Sea urchin granuloma of the skin. Arch. Dermatol..

[83] Moynahan E.J., Montgomery P.R. (1968). Echinoderm granuloma: A skin lesion resulting from injury by the spines of sea-urchins inhabiting temperate waters. A new mycobacterial infection. Br. J. Clin. Pract..

[84] Baden H.P. (1987). Injuries from sea urchins. Clin. Dermatol..

[85] Wörheide G., Dohrmann M., Erpenbeck D., Larroux C., Maldonado M., Voigt O., Borchiellini C., Lavrov D.V. (2012). Deep phylogeny and evolution of sponges (phylum Porifera). Adv. Mar. Biol..

[86] Gaino E., Manconi R., Pronzato R. (1995). Organizational plasticity as a successful conservative tactics in sponges. Anim. Biol..

[87] Sun W., Dai S., Jiang S., Wang G., Liu G., Wu H., Li X. (2010). Culture-dependent and culture-independent diversity of *Actinobacteria* associated with the marine sponge *Hymeniacidon perleve* from the South China Sea. Antonie van Leeuwenhoek.

[88] Izumi H., Gauthier M.E.A., Degnan B.M., Ng Y.K., Hewavitharana A.K., Shaw P.N., Fuerst J.A. (2010). Diversity of *Mycobacterium* species from marine sponges and their sensitivity to antagonism by sponge-derived rifamycin-synthesizing actinobacterium in the genus *Salinispora*. FEMS Microbiol. Lett..

[89] Tortoli E., Bartoloni A., Bozzetta E., Burrini C., Lacchini C., Mantella A., Penati V., Simonetti M.T., Ghittino C. (1996). Identification of the newly described *Mycobacterium poriferae* from tuberculous lesions of snakehead fish (*Channa striatus*). Comp. Immunol. Microbiol. Infect. Dis..

[90] Alderman D.J., Feist S.W., Polglase J.L. (1986). Possible nocardiosis of crayfish, *Austropotamobius pallipes*. J. Fish Dis..

[91] Plikaytis B.B., Plikaytis B.D., Yakrus M.A., Butler W.R., Woodley C.L., Silcox V.A., Shinnick T.M. (1992). Differentiation of slowly growing *Mycobacterium* species, including *Mycobacterium tuberculosis*, by gene amplification and restriction fragment length polymorphism analysis. J. Clin. Microbiol..

[92] Deepa P., Therese K.L., Madhavan H.N. (2005). Application of PCR-based restriction fragment length polymorphism (RFLP) for the identification of mycobacterial isolates. Indian J. Med. Res..

[93] Roth A., Fischer M., Hamid M.E., Michalke S., Ludwig W., Mauch H. (1998). Differentiation of phylogenetically related slowly growing mycobacteria based on 16S-23S rRNA gene internal transcribed spacer sequences. J. Clin. Microbiol..

[94] Lappayawichit P., Rienthong S., Rienthong D., Chuchottaworn C., Chaiprasert A., Panbangred W., Saringcarinkul H., Palittapongarnpim P. (1996). Differentiation of *Mycobacterium* species by restriction enzyme analysis of amplified 16S–23S ribosomal DNA spacer sequences. Tuberc. Lung Dis..

[95] Harvell C.D., Mitchell C.E., Ward J.R., Altizer S., Dobson A.P., Ostfeld R.S., Samuel M.D. (2002). Climate warming and disease risks for terrestrial and marine biota. Science.

[96] Brito-Morales I., Schoeman D.S., García Molinos J., Burrows M.T., Klein C.J., Arafeh-Dalmau N., Kaschner K., Garilao C., Kesner-Reyes K., Richardson A.J. (2020). Climate velocity reveals increasing exposure of deep-ocean biodiversity to future warming. Nat. Clim. Chang..

[97] Guschina I.A., Harwood J.L. (2006). Mechanisms of temperature adaptation in poikilotherms. FEBS Lett..

[98] Kim S.H., Shin J.H. (2017). Identification of nontuberculous mycobacteria using multilocous sequence analysis of 16S rRNA, *hsp65*, and *rpoB*. J. Clin. Lab. Anal..

